# The outcome of enhanced recovery after surgery vs. a traditional pathway in adolescent idiopathic scoliosis surgery: A retrospective comparative study

**DOI:** 10.3389/fsurg.2022.989119

**Published:** 2022-10-05

**Authors:** Hongtao Ding, Yong Hai, Li Guan, Yuzeng Liu, Aixing Pan, Bo Han

**Affiliations:** Beijing Chaoyang Hospital, Capital Medical University, Beijing, China

**Keywords:** enhanced recovery after surgery, adolescent idiopathic scoliosis, posterior spinal fusion, length of hospital stay, multimodal analgesia

## Abstract

**Objectives:**

The optimized enhanced recovery after surgery (ERAS) pathway for adolescent idiopathic scoliosis (AIS) patients has not been comprehensively described. The purpose of the study was to explore the feasibility and efficacy of an integral process of ERAS protocol in posterior spinal fusion (PSF) surgery for AIS patients without three-column osteotomy.

**Methods:**

Based on the inclusion and exclusion criteria, a total of 90 AIS patients who underwent PSF were enrolled in the study. Forty-five patients followed a traditional pathway (TP) perioperative care and 45 were treated with an ERAS protocol designed and implemented by a multidisciplinary team. Patient demographic, clinical information, surgical data, and radiographic parameters were collected and analyzed retrospectively.

**Results:**

There is no significant difference in age, gender, body mass index, preoperative hemoglobin level, Cobb angle, curve type, average correction rate, fusion segments, and screw number between ERAS group and TP group. Regarding the estimated blood loss (EBL), surgical duration, pain intensity, drainage duration, drainage volume, first ambulation time, postoperative length of stay (LOS), and the incidence of blood transfusion, they were significantly less in ERAS group than those of TP group.

**Conclusions:**

Based on our findings, we found that the implementation of a standard ERAS protocol in AIS correction surgery could result in less EBL, lower pain intensity, early ambulation, shorter LOS, and rapid rehabilitation. We recommend the widespread adoption of ERAS protocols in AIS surgery.

## Introduction

Adolescent idiopathic scoliosis (AIS) accounts for the largest population of all types of spinal deformity, most of which need a correction surgery to prevent deformity from deterioration, especially in one's teenage (1–3). In China, it is reported the prevalence of scoliosis is as high as 1.02% in the pre-high school population, with more than 10,000 surgeries performed per annum (4). Posterior spinal fusion (PSF) has been proven to be an effective method and the standard procedure for AIS correction according to the current study (5, 6). Despite the advantages in radiological parameters improvement, PSF also brings massive pain, great physical trauma, and psychological stress to such patients (5, 7). The concerning challenges for postoperative care of PSF remain adequate pain control, effective management of opioid-related side effects, and delayed mobilization. Besides, postoperative hemorrhage, infection, or procedure-related complications may postpone recovery after surgery, with overall complication rates averaging approximately 9%–15% (8, 9).

First introduced by Kehlet in 1997 (10), an enhanced recovery after surgery (ERAS) pathway has been implemented in various surgical settings and shown to safely decrease the postoperative length of hospital stay (LOS) by 2–3 days and in the complication rate by 30%–50% while improving the satisfaction and outcomes following surgery (11). ERAS protocols consist of a series of evidence-based approaches to perioperative care, with the aim of reducing surgical-stress responses, early mobilization, early oral nutrition, early removal of urinary catheters, and prevention of nausea and vomiting (12, 13).

However, the optimized enhanced recovery after surgery pathway for AIS patients has not been comprehensively described. The efficacy of the protocol should be verified. We are going to report the comparison of the outcomes between the ERAS pathway and the traditional method for AIS postoperative care.

## Methods

Ahead of the study, we received the approval of the ethics committee of BJCY hospital, CCMU (Approval number: ke2019–4-5), and it was performed in accordance with the Helsinki Declaration of 1964, and its later amendments. Informed consent was obtained from all individual participants included in the study.

Inclusion criteria were as follows: patients with AIS who underwent PSF without three-column osteotomy according to operation indications; aged 10–18 years; good physical and psychological status; no history of primary spinal surgery; and at least 1-year follow-up; curve correction achieved by pedicle screws and no procedure exposing the dura mater or performing three-column osteotomy. The PSF indications in AIS were spinal curvature >50° in those with a mature skeleton; or spinal curvature >45° in patients with an immature skeleton and orthotic management that did not prevent the curve from worsening (Cobb angle development >5° within 6 months). The exclusion criteria were: non-idiopathic scoliosis; history of spinal surgery; patients with hematologic diseases or preoperative hemoglobin (HB) level <100 g/L; those with missing data, and patients and families with poor compliance; other conditions that prevent compliance of the ERAS pathway.

We explored and started the ERAS protocol from 2018 to 2019, patients before this point underwent a traditional perioperative care method (traditional pathway group, TP), and those after received rapid recovery care (ERAS group). What should be noted, all patients in both groups underwent similar surgical procedures by the same surgical team. The PSF was achieved using the same pedicle screw-rod system. The surgical procedures were described as exposure of the spine from the skin to the periost, pedicle screws were placed using a standard technique. Facetectomy was performed to increase the spinal flexibility, improve the curve correction as well as facilitate spine fusion, rather than three-column osteotomy. Fusion was augmented using both autogenous and allogeneic bone grafts. Besides, complications were managed similarly and hospital discharge criteria were the same.

A standard ERAS protocol was designed and implemented by a multidisciplinary team comprising spine surgeons, anesthesiologists, nurses, a psychiatrist, and a nutritionist (the psychiatrist and nutritionist help to give a nutritional status evaluation and mental health assessment to optimize the status of patients) based on evidence-based elements and an understanding of rapid recovery principles. Before the protocol was developed, the traditional pathway of PSF perioperative care was executed by the same team, the comparison of procedures between the two groups is listed in Table 1 (14, 15). The ERAS protocol consisted of three components according to protocol order. The discharge guideline for the two groups is the same, namely, stable vital signs and good mental status, afebrile with no staining on the dressing, tolerable and reduced pain, a routine diet, independent of bowel movement, ambulating independently over 100 m without rest, and mastering the rehabilitation exercises independently.

**Table 1:** The protocol of ERAS method and traditional pathway.

Procedure	ERAS group	TP group
Preoperative preparation		
Communication and education	1. Tell the patients about the process, risks, and complications of anesthesia and surgery, to relieve the stress and anxiety from the unknown. 2. Tell the patients about the scheme and principle of ERAS protocol, including the diet, rehabilitation, pain management, and skin cleaning during the perioperative period, to increase compliance with program implementation. 3. Tell the patients about the discharge criteria and general information. 4. Tell the patients about the follow-up scheme, the approach, and the situation of readmissions.	General information about surgery, risks, complications, and rehabilitation.
Special exercise regarding surgery	Start from admission. 1. Start pulmonary function exercise through balloon blowing. 2. Start aerobic exercise by climbing the stairs. 3. Start flexibility exercise by spine extension strengthening and traction.	Not applicable.
Evaluation	1. General evaluation, including demographic characteristics, like weight, height, age, etc., vital signs, like heart rate, blood pressure, blood oxygen, etc. 2. Cardiopulmonary function evaluation. 3. Blood evaluation: coagulation function, electrolyte balance. 4. Nutritional status evaluation. 5. Pain intensity evaluation. 6. Self-function evaluation. 7. Mental health assessment.	1. General evaluation, including demographic characteristics, like weight, height, age, etc., vital signs, like heart rate, blood pressure, blood oxygen, etc. 2. Cardiopulmonary function evaluation. 3. Blood evaluation: coagulation function, electrolyte balance.
Intestinal preparation	1. Clear fluids up to 2 h and solids up to 6 h before induction of anesthesia. 2. Use of preoperative concentrated carbohydrate contained beverage routinely (or drink a 10% glucose 5 ml/kg). 3. Gastrointestinal motility drugs are used to treat abdominal distension after surgery.	No food or drink intake for 8 h before induction of anesthesia.
Intraoperative procedures		
Positioning	1. Pay attention to the chest and abdomen when placing, and reduce the abdominal pressure. 2. Apply elastic compress to skin contact area (shoulders, elbows, chest and lower ribs, anterior superior iliac spine, knees, ankle) to avoid skin damage, protect ulnar nerve, and common peroneal nerve.	General position.
Antibiotic prophylaxis	Antibiotic within 0.5 h of incision, additional antibiotic when the surgery duration exceeds every 3 h.	Same as ERAS.
Anesthesia	General anesthesia. 1. Induction stage based on propofol (2.5 mg/kg, i.v.), midazolam (1–2 mg, i.v.), sufentanil (0.1–0.5 mg/kg, i.v.), and rocuronium (0.6 mg/kg, i.v.). Avoid using inhalation agents and neuromuscular blockade. 2. Maintain stage, propofol (9–15 mg/kg, iv), remifentanil (0.2 μg/kg/min, i.v.).	General anesthesia. Medications rely on individual preference.
Pain management	Multimodal analgesia 1. COX-2 inhibitor (e.g., parecoxib, 40 mg, i.v.) and opioid (e.g., oxycodone, 0.1–0.2 mg/kg, i.v.), within 0.5 h of induction. 2. Maintenance, remifentanil (0.1–0.3 mg/kg/min, i.v. v.p.), dexmedetomidine (0.4 mg/kg/h, i.v. v.p.), and propofol (target-controlled infusion, 4–12 mg/kg/h). 3. Avoid neuromuscular blockade during surgery	Medications rely on individual preference.
Fluid management	Restricted target-oriental fluid therapy.	Medications rely on individual preference.
Temperature management	To maintain a core temperature of 36 °C 1. Fluid warming. 2. Airway humidification. 3. Underbody warm air blower. 4. Warming blanket. 5. Increasing OR temperature.	No precaution for hypothermia
Blood management	1. Controlled hypotensive anesthesia (mean arterial pressure 70–75 mm Hg). 2. Intraoperative cell salvage. 3. TXA (impaction dose 20 mg/kg before skin incision + infusion 10 mg/kg/h + 3 g TXA topical application). 4. Transfusion of blood products when hemoglobin <70 g/L.	Transfusion of blood products when hemoglobin <70 g/L.
Drainage	Subfascial drainage	Subfascial drainage
Surgical techniques	PSF, pedicle screw-rod system, MEPs and SEPs, ultrasonic osteotome.	PSF, pedicle screw-rod system, ultrasonic osteotome.
Postoperative care		
Pain management	Multimodal analgesia 1. Local subcutaneous was applied before skin closure with 0.75% ropivacaine (10 ml) + 0.9% saline (10 ml). 2. Patient-controlled analgesia pump: sufentanil (100 mg) + butorphanol (8 mg) + 0.9% saline, 100 ml totally. 3. COX inhibitor-2 (parecoxib, 40 mg, b.i.d., i.v.) from POD 1, until a favorable pain intensity but no more than 5 days. 4. Oral analgesics began on POD 2, celecoxib capsule (200 mg, b.i.d.) or etoricoxib tablets (120 mg, q.d.), or loxoprofen sodium tablets (60 mg, b.i.d.).	Medications rely on individual preference.
Intake management	1. Clear liquid allowed as requested and tolerated from 2 h postoperatively. 2. Soft diet was commenced 4–6 h as tolerated. 3. Normal diet was allowed on POD 1 if the patient has no PONV. 4. High-quality protein diet was advised from POD 1. 5. Folic acid tablets, iron ions, nourishing blood drink (Chinese medicine), etc., to improve hemoglobin levels.	1. No oral intake until 6 h postoperatively. 2. Liquid and soft diet started on 24–48 h as tolerated. 3. Normal diet started at least 48 h.
Anti-PONV therapy	1. Dual antiemetic prophylactic therapy (ondansetron, 4 mg + dexamethasone, 10 mg, i.v.). 2. Metoclopramide (10 mg, intramuscularly) if nausea and vomiting.	Metoclopramide (10 mg, intramuscularly) if nausea and vomiting.
Rehabilitation plan	1. Encourage mobilization and ambulation independence. 2. Removal of the catheter after anesthesia recovery. 3. Removal of subcutaneous drainage within 24 h (drainage <100 ml daily). 4. Wear customized brace as soon as ambulation within POD 30 days.	1. Early ambulation after removal of drainage. 2. Removal of the catheter after ambulation. 3. Maintain the subfascial drainage when the drainage <100 ml (at least 48 h postoperatively). 4. Ambulation on POD 3.

i.v., intravenous injection; COX-2, cyclooxygenase 2; i.v. v.p., intravenous pumping; OR, operative room; TXA, tranexamic acid; PSF, posterior spinal fusion; MEP, electric motor evoked potential; SEP, somatosensory evoked potential; b.i.d., twice daily; POD, postoperative day; PONV, postoperative nausea, and vomiting; ERAS, enhanced recovery after surgery; TP, traditional pathway.

### Outcome measures

Patient demographic, clinical information, surgical data, and radiographic parameters were collected retrospectively. The demographic information included the age, gender, and body mass index (BMI) of the patients. Clinical data, including preoperative and postoperative HB levels, postoperative pain intensity score (visual analog score, VAS), analgesic medicine use duration, drainage duration, first ambulation time, and LOS were documented. Radiological parameters in our study were preoperative and postoperative Cobb angle of the main curve, correction rate of the main curve, and curve type (Lenke classification for AIS). Surgical information including duration, estimated blood loss (EBL), instrumented levels, and screw numbers were extracted from the medical records. Postoperative complications and hospitalization of surgery were also analyzed.

### Statistical analysis

The SPSS version 18 software (IBM Corp., Armonk, NY, United States) was used to perform statistical analyses. Two-sample independent *t*-test was conducted to assess the differences of continuous variables with parametric data between the two cohorts. $\bar{\chi }$ and Fisher's exact test was used to analyze differences of categorical variables in outcome variables, where a *p* value of ≤0.05 was considered statistically significant.

## Result

### Demographic characteristics

A total of 90 AIS patients who underwent PSF were reviewed, with 45 in the ERAS group and 45 in the traditional group. There are four and five male patients in ERAS group and TP group, respectively, with a total average age of 15.36 ± 1.33 and 15.35 ± 1.53 years, respectively. The demographic characteristics of the patients are shown in Table 2. There is no significant difference in age, gender, and BMI between ERAS group and TP group. Regarding preoperative hemoglobin level, Cobb angle, and Lenke classification for AIS of curve type, the difference is not statistically significant.

**Table 2:** Demographic characteristics.

	ERAS group	TP group	*P* value
Sample size	45	45	—
Gender (M: F)	4:41	5:40	1.000
Age (y)	15.36 ± 1.33	15.35 ± 1.53	0.933
BMI (kg/m^2^)	21.04 ± 1.43	21.12 ± 1.26	0.772
Preoperative hemoglobin level (g/L)	115.29 ± 7.03	114.89 ± 6.14	0.774
Curve type (Lenke classification): (1:2:3:4:5:6)	2:17:18:5:2:1	1:16:16:6:5:1	0.868

BMI, body mass index; ERAS, enhanced recovery after surgery; TP, traditional pathway.

### Surgical characteristics of two groups

Both groups achieved outstanding deformity correction, with an average correction rate of more than 75%. The Cobb angle of the main curve was corrected from 89.20° ± 11.70° to 20.38° ± 7.16° in the ERAS group and from 85.27° ± 10.16° to 19.96° ± 4.68° in the TP group. Similar fusion segments and screws were employed in both ERAS and TP groups. However, the EBL and surgical duration in ERAS group were significantly less than those of TP group (*p* = 0.000 and 0.000). The detailed information was listed in Table 3.

**Table 3:** Surgical information of two groups.

	ERAS group	TP group	*P* value
Preoperative cobb of main curve (°)	89.20 ± 11.70	85.27 ± 10.16	0.092
Postoperative Cobb of main curve (°)	20.38 ± 7.16	19.96 ± 4.68	0.741
Correction rate (%)	77.46 ± 6.24	76.71 ± 4.25	0.507
Fusion segment	11.38 ± 1.80	11.16 ± 1.78	0.558
Screw number	22.62 ± 3.45	22.22 ± 3.44	0.583
Estimate blood loss (ml)	313.22 ± 39.73	402.89 ± 37.58	0.000
Surgical duration (min)	244.11 ± 26.46	264.33 ± 23.76	0.000

ERAS, enhanced recovery after surgery; TP, traditional pathway.

### Postoperative recovery characteristics

The postoperative hemoglobin in ERAS group (114.76 ± 6.74) was significantly higher (*p* < 0.001) than that of TP group (107.56 ± 6.46). The VAS for pain intensity of postoperative day (POD) 1 and POD 3 in the ERAS group was 3.89 ± 0.91 and 2.04 ± 0.64, both of which were significantly lower than those of the TP group (4.80 ± 0.84 for POD 1 and 3.04 ± 0.74 for POD 3). In terms of analgesic medicine applied duration, it was 2.36 ± 0.77 days in ERAS group and 4.51 ± 0.87 days in TP group, which exhibited statistical difference. Drainage duration and volume in the ERAS group were 1.38 ± 0.49 days and 61.13 ± 11.05 ml, both were less than those of the TP group (*p* < 0.001). The first ambulation time for patients in ERAS group is 2.27 ± 0.58 days, which was shorter than 4.96 ± 0.74 days for patients in TP group. The postoperative LOS in the ERAS group was significantly less than in the TP group (4.64 ± 0.86 vs. 6.22 ± 0.97). The allogeneic blood transfusion happened in 3 cases (6.67%) in ERAS group and 12 cases (26.67%) in TP group, which was significantly higher in TP group.

Of the 45 patients in ERAS group, 24 patients returned home on POD 4 (53.33%), 16 returned home on POD 5 (35.56%), 2 returned home on POD 6 (4.44%), and 3 returned home on POD 7 (6.67%). Of the latter 5 patients, 2 had a postoperative fever, 1 for wound infection, and 2 for nausea and vomiting, which were all postoperative complications in the ERAS group. In TP group, 10 patients returned home on POD 5 (22.22%), 21 returned home on POD 6 (46.67%), 9 returned home on POD 7 (20.00%), 4 returned home on POD 8 (8.89%), 9 returned home on POD 9 (2.22%). The complications in the TP group consisted of 4 cases of fever, 4 cases of wound infection, and 5 cases of nausea and vomiting. The overall postoperative recovery characteristics were shown in Table 4.

**Table 4:** Postoperative recovery characteristics and early complications of two groups.

	ERAS group	TP group	*P* value
Postoperative recovery characteristics			
POD 1 hemoglobin level (g/l)	114.76 ± 6.74	107.56 ± 6.46	0.000
VAS of POD 1	3.89 ± 0.91	4.80 ± 0.84	0.000
VAS of POD 3	2.04 ± 0.64	3.04 ± 0.74	0.000
Analgesic medicine (day)	2.36 ± 0.77	4.51 ± 0.87	0.000
Drainage duration (day)	1.38 ± 0.49	3.80 ± 0.73	0.000
Drainage volume (ml)	61.13 ± 11.05	433.33 ± 107.66	0.000
First ambulation time (day)	2.27 ± 0.58	4.96 ± 0.74	0.000
Postoperative LOS (day)	4.64 ± 0.86	6.22 ± 0.97	0.000
Early complications			
Fever	2	4	0.677
Wound infection	1	4	0.361
Nausea and vomiting	2	5	0.434
Allogeneic blood transfusion	3	12	0.007

POD, postoperative day; VAS, visual analog score; LOS, length of stay; ERAS, enhanced recovery after surgery; TP, traditional pathway.

## Discussion

The method of enhanced recovery after surgery was introduced 25 years ago by Kehlet (10). The components of the optimal idea were a series of evidence-based protocols of perioperative care to reduce surgical-stress responses and provide rapid rehabilitation for patients after operation. It has been reported and validated to be effective in various surgical procedures to accelerate postoperative recovery, which is an ideal concept for postoperative rehabilitation of posterior spinal fusion (11, 12, 16).

PSF for scoliosis is known as long duration, traumatic, heavy bleeding, and high risk of neurologic complications. The adoption of ERAS in scoliosis surgery has been explored before. Fletcher et al. reported a novel pathway for patients with AIS undergoing PSF that shortened the LOS without increasing the incidence of complications in 2014 (17). In 2021, Fletcher et al. found patients managed with both an ERAS pathway and a traditional pathway could have a rapid return to normalcy through a prospective dual-center study with 280 patients, but it was shown a 55% less LOS and a significantly less length of surgery and EBL in the ERAS group (18). Rather than a comprehensive and overall protocol for ERAS method, previous studies focus mainly on individual components of ERAS. An optimized ERAS pathway has been lacking in this setting. Thus, we seek to explore the feasibility and efficacy of an integral process of ERAS in PSF for AIS patients.

Based on the advanced experience of previous studies and the characteristics of young patients, we set a multidepartment protocol for AIS surgery including spine surgeon, nurse, anesthetist, psychiatrist, and nutritionist (19, 20). For preoperative preparation, the main goals are performing comprehensive assessment, optimizing the nutritional, psychological, and cardiopulmonary function status, alleviating the tension between patients and their families, and making good communication between doctors and patients. Besides scoliosis correction, it is of great importance to minimize surgical trauma, reduce blood loss, and maintain optimal blood pressure and temperature during operation. Postoperatively, performing satisfied pain management, accelerating rehabilitation, and preventing complications rank first. Nevertheless, spine deformity in adolescents affects the psychological status adversely. It is reported that 40% of AIS patients suffered from solitude and depression during and after treatment (21, 22). Deformity correction was reported to improve the physical and mental health of patients with AIS (23). Spine surgeons should keep aware that preoperative education could contribute to increasing self-confidence and reducing stress to improve patients' psychosocial status (24).

### Intraoperative procedure to reduce the EBL

Some measures taken during operation to minimize the surgical trauma and reduce the blood loss were controlling the lowering of blood pressure and tranexamic acid (TXA). The efficacy of TXA to minimize blood loss in AIS surgery has been explored and verified without increasing the risk of deep vein thrombosis (25, 26). The comprehensive studies illustrated that the application of TXA could reduce total blood loss perioperatively and result in a higher hemoglobin level in patients undergoing spinal surgery (27, 28). In our study, the combination of TXA and controlled hypotension result in significantly less EBL and drainage volume, a higher postoperative hemoglobin level, and a lower incidence of blood transfusion in the ERAS group compared to the TP group.

### Pain management

Postoperative pain management posed great challenges for AIS surgery. In addition to improving the quality of recovery, effective pain management reduces the patient's stress response, facilitates ambulation, and accelerates postoperative rehabilitation (29). Therefore, the significant role of multimodal analgesia is emphasized in all ERAS society guidelines (30). In the present study, the VAS of POD 1 and POD 3 in the ERAS group were significantly lower and the duration of analgesic medicine in the ERAS group was notably shorter than those of the TP group, which should be attributed to the effects of multimodal analgesia. The pain management for patients in ERAS group consisted of an incision infiltration of 0.375% ropivacaine, application of a patient-controlled analgesia pump (sufentanil + butorphanol), and COX-2 inhibitors medicine, which lead to both analgesia maintenance and reduced consumption of opioids (31).

### Reduction of length of stay

Length of stay (LOS) is the indicator of better care, reducing potential medical complications, and rapid rehabilitation. The postoperative LOS in our study for patients in ERAS group is significantly shorter than TP group. The reduction in LOS of 1–2 days is similar to the previous studies (20). The improvement could attribute to the following reasons: optimization of the nutritional status, reduction in EBL during operation, successful postoperative pain management, and acceleration of rehabilitation.

Early ambulation, less complication, and length of stay are the goals of ERAS concept. In ERAS group, the average time of the first ambulation was 2.27 ± 0.58 days, significantly shorter than 4.96 ± 0.74 days in TP group. Satisfied pain management by multimodal analgesia helped to reduce the bedtime before getting to walk, which also contributed to starting a chain reaction to reduce nausea and vomiting and rapid recovery (32). However, the difference in complication incidence between ERAS and TP groups showed no statistical significance.

### Limitations

Our study has limitations. Above all, it is a retrospective study with a small sample size within a single institution, which discounts the persuasive power of the conclusions. In addition, the surgeries were performed by a single surgeon, and as time goes by, the technique of surgery and skills proficiency might be a confounding factor to the outstanding results in the ERAS group. Therefore, a prospective randomized controlled study in multicenter is needed to verify the efficacy of the proposed comprehensive ERAS protocol.

## Conclusions

Based on our findings, we found that the implementation of a standard ERAS protocol in AIS correction surgery could result in less EBL, lower pain intensity, early ambulation, shorter LOS, and rapid rehabilitation. We recommend the widespread adoption of ERAS protocols in AIS surgery.

## Data Availability

The raw data supporting the conclusions of this article will be made available by the authors, without undue reservation.
